# Self-recovery reversible image watermarking algorithm

**DOI:** 10.1371/journal.pone.0199143

**Published:** 2018-06-19

**Authors:** Zhengwei Zhang, He Sun, Shangbing Gao, Shenghua Jin

**Affiliations:** 1 Faculty of Computer and Software Engineering, Huaiyin Institute of Technology, Huai’an, Jiangsu, China; 2 College of Command Information System, PLA University of Science and Technology, Nanjing, Jiangsu, China; King Saud University, SAUDI ARABIA

## Abstract

The integrity of image content is essential, although most watermarking algorithms can achieve image authentication but not automatically repair damaged areas or restore the original image. In this paper, a self-recovery reversible image watermarking algorithm is proposed to recover the tampered areas effectively. First of all, the original image is divided into homogeneous blocks and non-homogeneous blocks through multi-scale decomposition, and the feature information of each block is calculated as the recovery watermark. Then, the original image is divided into 4×4 non-overlapping blocks classified into smooth blocks and texture blocks according to image textures. Finally, the recovery watermark generated by homogeneous blocks and error-correcting codes is embedded into the corresponding smooth block by mapping; watermark information generated by non-homogeneous blocks and error-correcting codes is embedded into the corresponding non-embedded smooth block and the texture block via mapping. The correlation attack is detected by invariant moments when the watermarked image is attacked. To determine whether a sub-block has been tampered with, its feature is calculated and the recovery watermark is extracted from the corresponding block. If the image has been tampered with, it can be recovered. The experimental results show that the proposed algorithm can effectively recover the tampered areas with high accuracy and high quality. The algorithm is characterized by sound visual quality and excellent image restoration.

## 1 Introduction

Image content integrity is of great significance for special types of images (military images, medical images, legal images, etc.) since their important texture details contain some important information, such as the strike targets in military images, regions of interest in medical images, and evidence in legal images. A change in the content of an image can easily affect the outcome of the war, the health of the patient, or the notary of the law. With the rapid development of information technology, the application of digital images has become increasingly important, and image processing tools are correspondingly powerful. Accordingly, the authenticity and integrity of image contents are paramount. However, most of the watermarking algorithms can only be implemented based on image authentication or tamper localization [1]. People are eager to restore tampered areas and repair damaged areas automatically to facilitate the recovery of original image content. To realize image self-recovery, watermark data must be associated with the original image content. A destroyed original image can be restored using the watermark data hidden in the image. Although self-recovery image authentication algorithms [2, 3] have achieved remarkable research results, they also face many practical problems. In this paper, several self-recovery image authentication algorithms are analyzed, and a self-recovery reversible image watermarking algorithm is proposed.

Fridrich et al. [4] put forward a kind of self-embedding algorithm for the first time. The discrete cosine transform (DCT) coefficients of image blocks are quantized and then embedded into the least significant bit (LSB) of the offset block. Results revealed that when the tamper area is smaller, the recovery rate is higher. This embedded idea based on independent blocks can effectively resist vector quantization (VQ) attacks and collusion attacks, thereby improving the security of the algorithm to some extent. A defect of the method, however, is the fixed offset block mapping relationship that allows attackers to collect watermark information by estimating the offset after testing a number of images, diminishing the practicality of the algorithm. When the tampered area is larger, the location precision of the algorithm decreases. In light of this, literature [5, 6, 7] quantified and coded the DCT coefficients through analysis of DCT coefficient characteristics, thus improving the algorithm security to some extent. Yet the tamper localization accuracy and recovery quality need to be further improved. In [8], the image blocks are divided into smooth blocks and complex blocks by encoding nonsubsampled Contourlet transform (NSCT) coefficients with sound embedded quality and recovery quality. Because an image after NSCT is the same size as the original, image restoration possesses some advantages.

Pan et al. [9] proposed a fragile watermarking algorithm for image content recovery. They extracted the characteristic values of blocks based on the MD5 digital signature algorithm to generate watermarks. In the recovery system, the recovery watermark is derived from the data of the compressed original image; data are then embedded into the LSB image for self-recovery. This algorithm is simple and highly secure, which can repair the tampered areas and resist collage attacks effectively. Liu et al. [10] proposed a watermarking algorithm based on the hierarchical structure. In this approach, the watermark information is embedded into the 2-bit LSB original image. The authentication watermark is composed of parity-check codes and the average gray value between blocks; the recovery watermark is the average gray value of the torus self-isomorphic mapping block. The three-layered structure is found to improve the detection rate and recovery quality. In [11], a reversible information hiding algorithm combined reference bits and check bits into images. For small tampered areas, the original data can be recovered effectively through distortion-free data embedding, but the recovery distortion is more serious when the tampered area is greater than 4%. In [12], algorithm security is enhanced by combining the iterative pixel embedding mechanism with the block embedding mechanism. Compared with the traditional algorithm, the proposed algorithm can effectively detect and properly restore changes in image size caused by edge shear. However, the recovery quality is affected when the tampered area is large (i.e., more than 5%).

Many algorithms have been used to embed redundant information (e.g., DCT coefficients, wavelet transform coefficients, NSCT transform coefficients, and the mean) to achieve a certain degree of recovery; nevertheless, recovery quality is limited when the tampered area is too large. Therefore, to solve the problem of limited data embedding space, one must determine the real content or important information that can be captured in the image. Using an approach technology requiring less data to generate image recovery information should greatly improve recovery quality. In addition, the existing self-recovery watermarking algorithm is a traditional digital watermarking algorithm and thus performs worse in high quality recovery of the original carrier image, rendering high integrity in some types of images impossible.

A reversible image watermarking algorithm based on self-recovery is proposed in this paper. Firstly, the original image is divided into homogeneous blocks and non-homogeneous blocks through multi-scale decomposition, and the feature information of each block is calculated as recovery watermark information. Then, the original image is divided into 4×4 non-overlapping blocks that are then classified into smooth blocks and texture blocks according to image textures. Finally, the recovery watermark generated by the homogeneous blocks and error-correcting codes is embedded into the corresponding smooth block by mapping; watermark information generated by the non-homogeneous blocks and error-correcting codes is embedded into the corresponding non-embedded smooth block and the texture block using the same method. The correlation attack is detected by invariant moments when the watermarked image is attacked. To determine whether a sub-block has been tampered with, its feature is calculated by multi-scale decomposition and the recovery watermark is extracted from the corresponding block. If it has been tampered with, it will be recovered.

## 2 Technical background

### 2.1 Ethics statement

In this paper, all study images were derived from http://sipi.suc.edu/database. These images can be used under the public platform. Written informed consent was obtained from all enrolled subjects prior to the study. The individual in this manuscript has given written informed consent (as outlined in PLOS consent form) to publish these case details. Furthermore, we guaranteed that the privacy right of each subject was completely observed.

### 2.2 Image normalization

In this paper, a self-recovery reversible watermarking algorithm is proposed to embed watermark information into the original image for better self-recovery. After embedding recovery watermark information, the recovery authentication watermark is extracted from the attacked image in accordance with the previous algorithm for image partitioning, feature extraction, or tampering localization detection when the image is attacked by rotation, scaling, translation, and other related operations, which may be very inaccurate. Hence, an image should be normalized before a watermark is embedded and extracted.

The image normalization algorithm based on image features has parameters such as image rotation, translation, and scaling. The geometric transformation of an image is carried out as per a standard form. If a watermark is embedded into a normalized image, it can guarantee the invariance of the area where the image has sustained geometric attacks to ensure the robustness of the embedded watermark against such geometric attacks. To simplify the normalization process, the affine transformation matrix is decomposed into three simple change forms for matrix multiplication.

$$\left(\begin{array}{c} x_{n}\\ y_{n} \end{array}\right)=\left(\begin{array}{cc} \cos\phi & \sin\phi \\ -\sin\phi & \cos\phi \end{array}\right)\left(\begin{array}{cc} \alpha & 0\\ 0 & \delta \end{array}\right)\left(\begin{array}{cc} 1 & \beta \\ 0 & 1 \end{array}\right)\left(\begin{array}{c} x\\ y \end{array}\right)$$ (1)

where *α*,*β*,*δ* ∈ *R*, *ϕ* ∈ (0,*π*]. From right to left, respectively, the three transformation matrices are shear, scaling, and rotation and translation operations.

### 2.3 Multi-scale decomposition

An image *F* is divided into multiple scales from global to local [13], and sub-image blocks *F*_*i*, *j*_ are obtained at different scales, where *i* = 1, 2, …, *d* is denoted as the segmentation scale; *j* = 1, …, 4^*i*-1^ is denoted as the number of sub-blocks at each scale. The segmentation scale is constrained by image size limitations.

**Fig 1:**
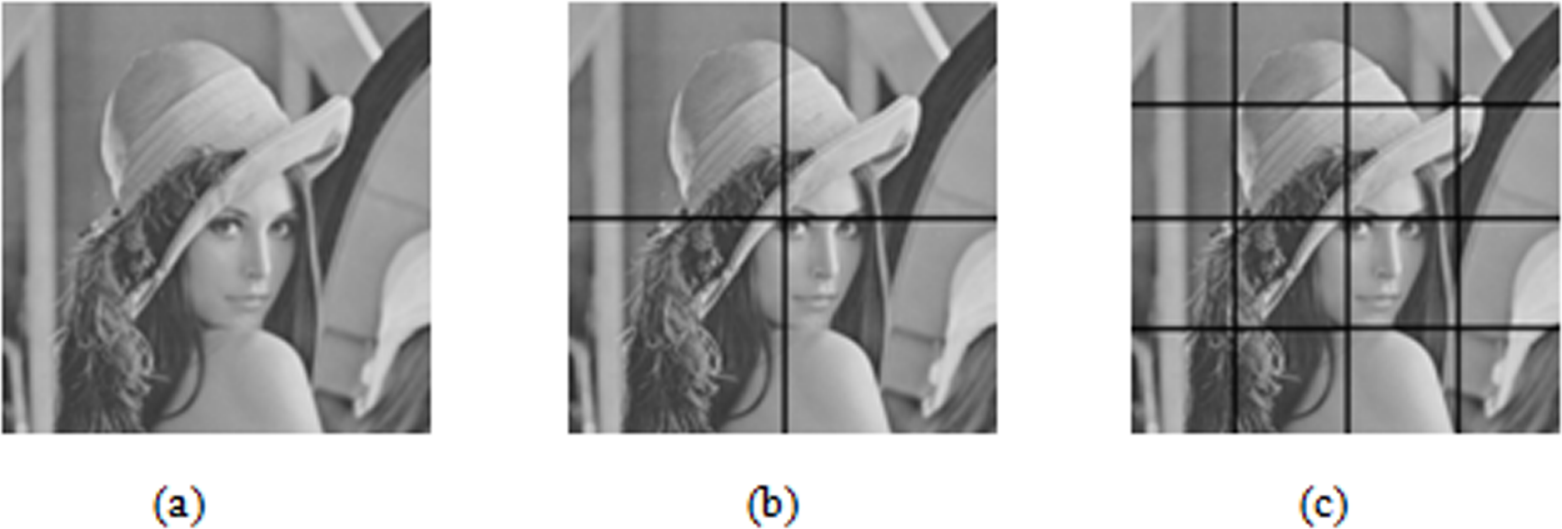
Multi-scale segmentation of *Lena* image. (a) represents the 1^st^ scale. (b) represents the 2^nd^ scale. (c) represents the 3^rd^ scale.

The segmentation method is shown in Fig 1. From left to right, segmentation occurs from the 1^st^ to 3^rd^ scales. The whole image is the sub-blocks *F*_1,1_ obtained after being decomposed at the 1^st^ scale, and then the whole image is divided into four sub-blocks (*F*_2,1_, *F*_2,2_, *F*_2,3_, and *F*_2,4_), each of which is called the second scale sub-block and is further divided until reaching the maximum scale set.

Given that traditional multi-scale decomposition is also a form of fixed-sized block decomposition, this paper improves multi-scale decomposition.

The improved image multi-scale decomposition is used to divide a rectangular image into four equal-sized square blocks and then determine whether these four blocks meet the homogeneity criterion. If so, then the current block either remains unchanged; if not, it will continue to be decomposed into four square blocks to determine whether they can meet the criterion until all blocks meet the criterion. The decomposition criterion can be expressed as:

$$\left|p_{i}-p_{ave}\right|>(g_{l}-1)\times \gamma$$ (2)

**Fig 2:**
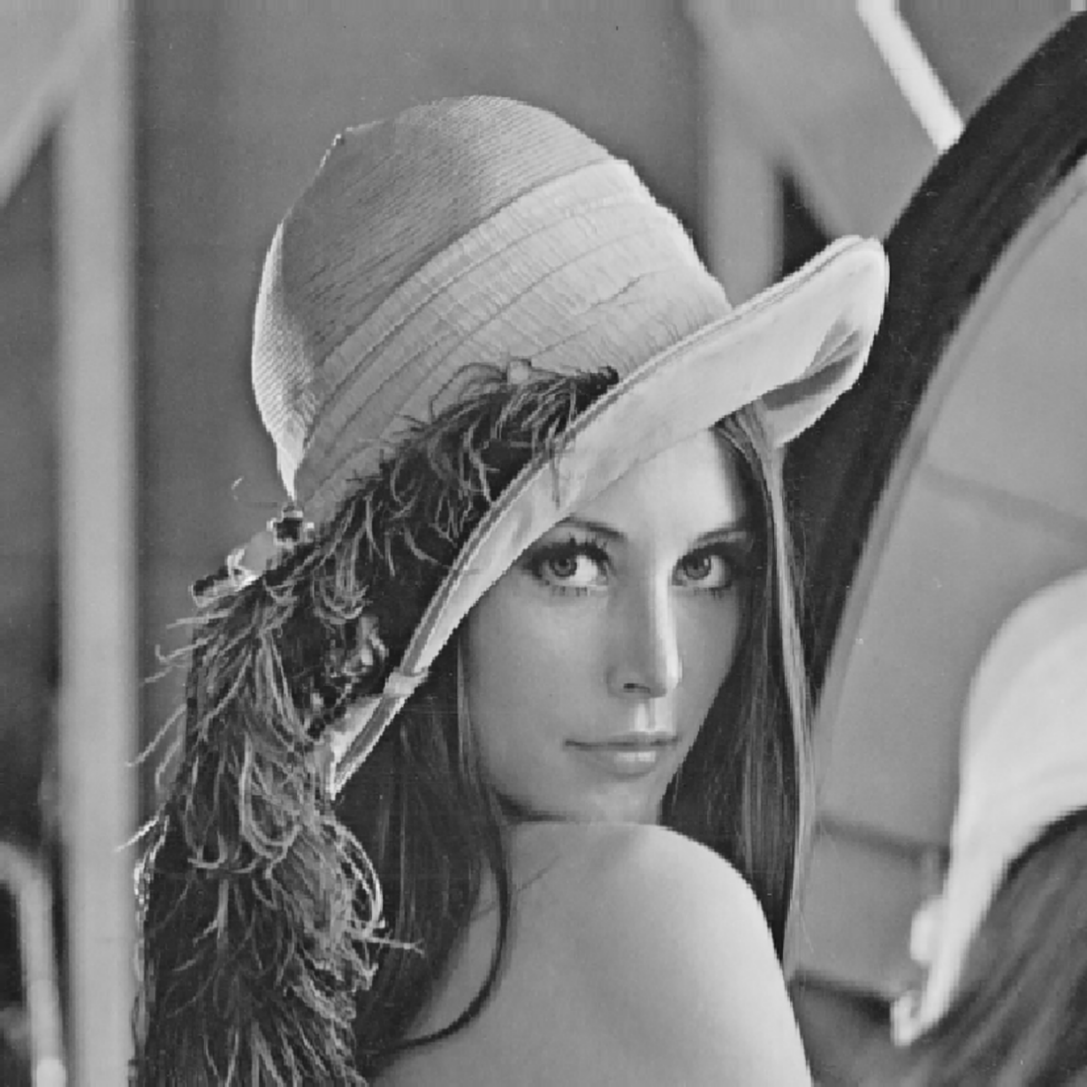
Original image.

**Fig 3:**
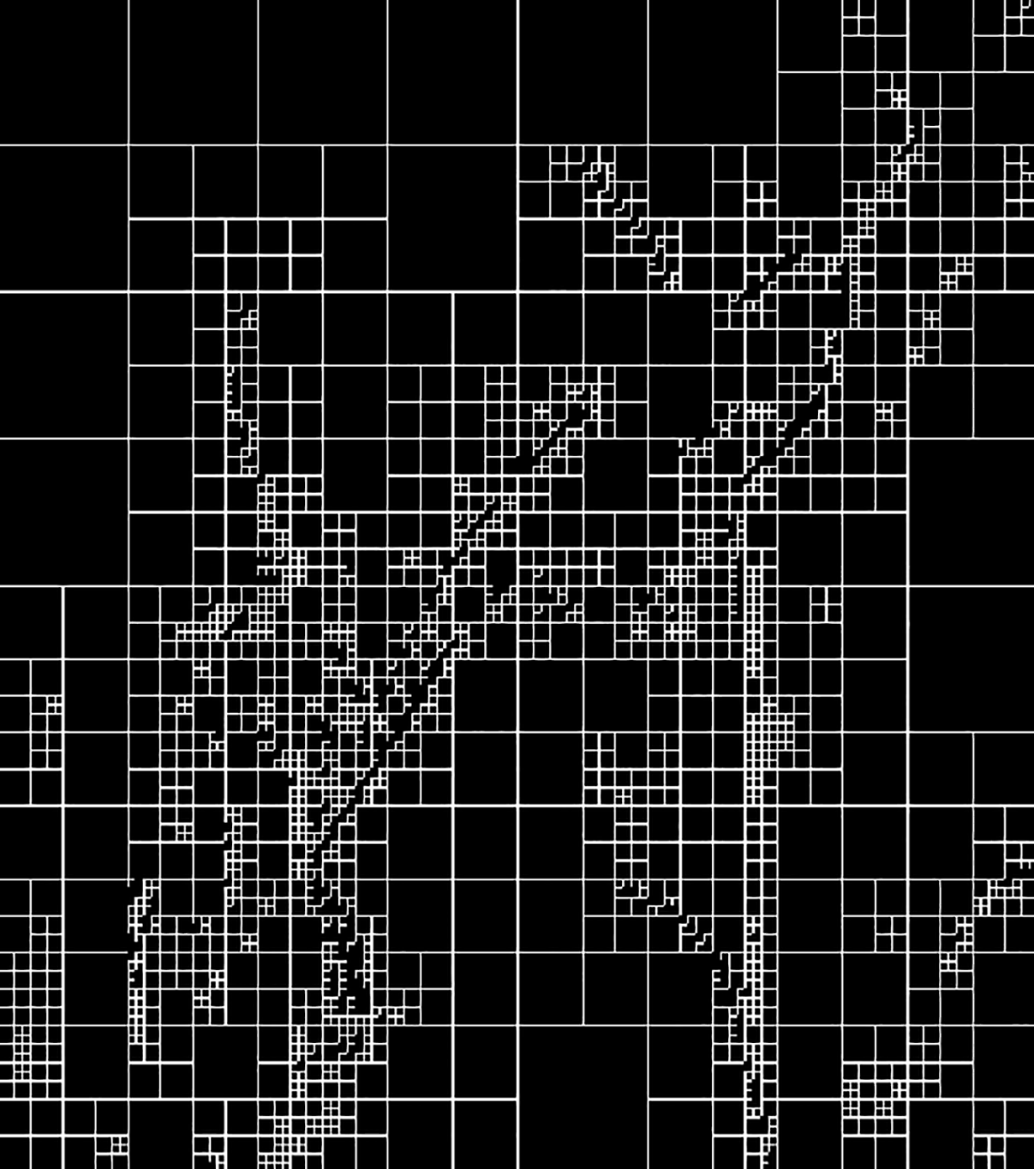
The generated block map after modified multi-scale decomposition.

In Formula (2), *p*_*i*_ and *p*_*ave*_ represent the gray value of any pixel and the average gray value of all pixels in a square block, respectively; *g*_*l*_ is the gray level of a pixel; and *γ* is a decimal in the range of [0,1]. This criterion requires the block to be further divided when the absolute value of the difference between the gray value of any pixel and the average gray value of all pixels in the square block is greater than (*g*_*l*_ −1) × *γ*. Please see Figs 2 and 3 for details.

Per the block division method, images are classified into unfixed sizes. Based on the division results, the decomposed image blocks have high homogeneity pixels suitable for lossless watermark embedding. As specified in the algorithm, the minimum block size is 4×4.

After multi-scale decomposition, the size of each image block is 2^2+*n*^×2^2+*n*^, *n*∈{0,1,2,…,7}. Each block size can be converted into a binary form. Each decomposed sub-block is coded by the sub-block size. Please see Table 1:

**Table 1:** Scale coding of image block after multi-scale decomposition.

Size / pixel	code	Size / pixel	code
4×4	000	64**×**64	100
8×8	001	128**×**128	101
16×16	010	256**×**256	110
32×32	011	512**×**512	111

After multi-scale decomposition, the obtained image sub-blocks are sorted (from top to bottom and left to right) and the scale information of each sub-block is recorded sequentially based on the ordering result, thereby constituting decomposition information *q* of the original image. Given a small number of decomposed large-sized blocks, Huffman encoding [14] can be used to further reduce the length of the image decomposition information, denoted as Huf (*q*).

To ensure the security of the algorithm, the length and encoding table of the parameter Huf (*q*) are sent to the recipient in the form of a secret key.

### 2.4 BCH encoding

The recovery watermark must be coded using error-correcting codes prior to embedding, after which it can be embedded into the original image. The recovery watermark will change when a tampering event occurs. The main information of the image blocks will also change as the recovery watermark information changes, further affecting the original image content. An error-correcting code is employed to ensure the correctness of the recovery watermark at the expense of effective embedding capacity.

A parity-check code is one of the simplest data check codes [15]; its code distance is equal to 2, which can detect an error (or odd bit error) but can neither locate it nor detect a digital error.

Hamming codes can correct one error encoding [16]. They use *k* bits to represent information bits and add *r* redundancy bits to constitute a *n* = *k* + *r* bits code word. Then, the *r* correction factors produced by the *r* supervisory relationship are used to distinguish the error-free area and a dislocation of *n* different locations in the code word. The following relationship must be satisfied:

$$2^{r}\geq k+r+1\;\mathrm{or}\;2^{r}\geq n+1$$

The BCH code is a cyclic code [17] with high correction ability, making it one of the best linear block codes currently available. The BCH code was proposed by Hocquenghem in 1959 and then by Bose and Chandhari in 1960. The BCH code is used to correct a number of random-error-correcting cyclic codes and can be described using the roots of generated polynomial *g*(*x*). This paper assumes any finite field *GF*(*q*) and its extended domain *GF*(*q* ^m^) are given, where *q* is a prime or a power of a prime and *M* is a positive integer. If the code element is a symbol taken from *GF*(*q*), then the root set *R*_1_ generated by its polynomial *g*(*x*) contains the following *δ*-1 consecutive roots:

$$a^{m_{0}},\;a^{m_{0}+1},\;a^{m_{0}+2},\;\ldots ,\;a^{m_{0}+\delta -2}$$

The cyclic code generated by *g*(x) is called the *q* binary BCH code, namely the *q* element BCH code, where $a^{m_{0}+i}\in GF(q^{m})$ and *m*_0_ is an arbitrary integer. When *q* = 2, two-element BCH codes can be acquired.

*β* = *α*^*l*^ ∈ *GF*(2^*m*^) is assumed. *l* is an arbitrary integer, and *α* is the primitive element of *GF*(2^*m*^). If *V* represents the cyclic codes from *GF* (2) of code length *n*, then the root set *R*_2_ of the generated polynomial *g*(*x*) contains the following 2*t* consecutive roots:

$$\beta ,\;\beta^{2},\;\beta^{3},\ldots ,\;\beta^{2t}$$

The cyclic code generated by *g*(x) is called a two-element BCH code, the supervision matrix of which can be expressed as:

$$H=\left[\begin{array}{ccccc} \beta^{n-1} & \beta^{n-2} & \cdots & \beta & 1\\ {\left(\beta^{3}\right)}^{n-1} & {\left(\beta^{3}\right)}^{n-2} & \cdots & \beta^{3} & 1\\ \cdots & \cdots & \cdots & \cdots & \cdots \\ {\left(\beta^{2t-1}\right)}^{n-1} & {\left(\beta^{2t-1}\right)}^{n-2} & \cdots & \beta^{2t-1} & 1 \end{array}\right]$$ (3)

The *m* in BCH (*m*, *n*, *t*) denotes the total encoding bits, including *n* information bits and *m*-*n* monitoring bits. Use of this encoding information can resist the error of *T* bits information per *m* bits information. BCH (*m*, *n*, *t*) takes various formats, such as BCH (7,4,1), BCH (7,1,3), BCH (15,11,1), BCH (15,7,2), BCH (15,5,3), BCH (31,6,7), and BCH (63,7,15). The efficiency of encoding and error correction must be considered when using BCH. The error correction capability of BCH (63,7,15) is strongest; every 7 effective information bits need to use 56 monitoring bits and correct random errors under 15 bits per 63 bits encoding information through a large number of monitoring bits. BCH (15,11,1) is an encoding method in which the error correction ability is relatively weak but the encoding efficiency is high. The method proposed in this paper is mainly intended for tamper detection and recovery of images with little tampering, including no strong requirement for error correction capability. Yet to preserve as much image information as possible, encoding requires high efficiency. Therefore, the recovery watermark information is encoded by choosing BCH (15,11,1), which possesses high efficiency and low error-correcting capabilities, to resist an error rate of 6.67% of the random errors. Due to the BCH code, the error-correcting capability is relatively stable in both low-noise and high-noise channels. As such, in this paper, the BCH code is used to increase the robustness of the watermark.

### 2.5 Invariant moments

The watermark synchronization error caused by geometric attacks can be minimized using the geometrically invariant features of the image to identify the accurate location of watermark embedding and detection. Zernike moments are a set of orthogonal moments with a characteristic of rotation invariance [18]. As an ideal image feature descriptor, the set is insensitive to noise and can be expressed simply. It has been widely used in pattern recognition, image understanding and other applications. The polynomials of Zernike moments are defined as follows:

$$V_{nm}(x,y)=V_{nm}(r,\theta )=R_{nm}(r)•e^{jm\theta }$$ (4)

where $r=\sqrt{x^{2}+y^{2}}$, *θ* = arctan(*y*/*x*). *n* is a non-negative integer, and *m* is an integer that meets *n*−|*m*| as an even and |*m*| ≤ *n*. *R*_*nm*_(*r*) is the radial polynomial of Zernike moments.

$$R_{nm}(r)=\sum_{s=0}^{(n-\left|m\right|)/2}{\left(-1\right)}^{s}•\frac{(n-s)!}{s!\left(\frac{n+\left|m\right|}{2}-s\right)!\left(\frac{n-\left|m\right|}{2}-s\right)!}$$ (5)

Then, the *n*-order Zernike moments with cycle index *m* are:

$$Z_{nm}=\frac{n+1}{\pi }\sum_{x}\sum_{y}f(x,y)•V_{nm}^{*}(x,y)=\frac{n+1}{\pi }\sum_{r}\sum_{\theta }f(r\cos\theta ,r\sin\theta )•R_{nm}(r)•e^{jm(-\theta )}$$ (6)

(1) Rotation detection

If the image is rotated *α* counterclockwise, the relationship between before and after the Zernike moments rotation is as follows:

$$Z_{nm}^{'}=Z_{nm}•e^{jm\alpha }$$ (7)

The rotation angle *α* can be calculated using the following formula:

$$\alpha =\frac{\arg(Z_{nm}^{'})-\arg(Z_{nm})}{m},m\neq 0$$ (8)

(2) Scaling attack

To make *f*(*x*/*a*,*y*/*a*) denote the image after scaling *a* times with the original image, the relationship between before and after the Zernike moments scaling image is as follows:

$$\left|Z_{nm}^{'}\right|=a^{2}\;\left|Z_{nm}\right|$$ (9)

The scaling factor can be obtained by the following formula:

$$a=\sqrt{\left|Z_{nm}^{'}\right|/\left|Z_{nm}\right|}$$ (10)

(3) Rotation attack

To make $Z_{nm}^{\left(hf\right)}$ denote the Zernike moments of image *f*^(*hf*)^(*x*,*y*) after horizontal rotation, $Z_{nm}^{\left(vf\right)}$ represents the Zernike moments of image *f*^(*vf*)^(*x*,*y*) after vertical rotation. $Z_{nm}^{*}$ represents the complex conjugate of *Z*_*nm*_.

$$Z_{nm}^{\left(hf\right)}=\frac{n+1}{\pi }\sum_{x}\sum_{y}f^{hf}(x,y)•V_{nm}^{*}(x,y)=\frac{n+1}{\pi }\sum_{x}\sum_{y}{\left(-1\right)}^{m}f(x,y)•R_{nm}(r)•e^{jm\theta }={\left(-1\right)}^{m}Z_{nm}^{*}$$ (11)

Similarly,

$$Z_{nm}^{\left(vf\right)}=\frac{n+1}{\pi }\sum_{x}\sum_{y}f^{vf}(x,y)•V_{nm}^{*}(x,y)=\frac{n+1}{\pi }\sum_{x}\sum_{y}{\left(-1\right)}^{m}f(x,y)•R_{nm}(r)•e^{jm\theta }={\left(-1\right)}^{m}Z_{nm}^{*}$$ (12)

Formula (11) shows that the Zernike moments of the image after the horizontal flip can be either $Z_{nm}^{\left(hf\right)}=Z_{nm}^{*}$ (*m* is even) or $Z_{nm}^{\left(hf\right)}=-Z_{nm}^{*}$ (*m* is odd). Similarly, Formula (12) shows the Zernike moments of the image after the vertical flip are represented as $Z_{nm}^{\left(vf\right)}=-Z_{nm}^{*}$ (*m* arbitrary integer).

## 3 Algorithm design

The proposed self-recovery reversible image watermarking algorithm mainly includes three stages: recovery watermark generation, recovery watermark embedding, tamper localization and recovery of the watermarked image.

### 3.1 Recovery watermark generation

To recover the tampered watermarked image, the image texture statistical properties are used to generate the recovery watermark. Essentially, the original image is divided into homogeneous blocks and non-homogenous blocks by multiple scales. In homogeneous blocks, the pixel value averaging algorithm is used to extract the block feature as the recovery watermark of this block. Non-homogenous blocks are further divided into non-overlapping blocks, and the feature of each sub-block is generated as the recovery watermark. The generated recovery watermark is strung together in a series and converted to a binary number. This binary string is the recovery watermark, and the specific operation is as follows:

For homogeneous blocks, the average value of all pixels therein is calculated as the recovery watermark of that homogeneous block. Assuming the size of a divided homogeneous block is *N*×*N*, the recovery watermark is denoted using the 8-bit binary string F_*i*_ = {*f*_*i*1_, *f*_*i*2_, …, *f*_*i*8_}.

$$f_{i1}∼f_{i8}={\left(\frac{1}{N\times N}\sum_{i=1}^{N}\sum_{j=1}^{N}x_{i}{}_{j}\right)}_{B}$$ (13)

where (·)_B_ represents an integer binary encoding.

This paper assumes the minimum block size to be 4×4 after multi-scale decomposition, possibly containing two cases: homogeneous blocks and non-homogenous blocks. For the homogeneous block, its average value must be computed according to the above homogeneous block method.

**Fig 4:**
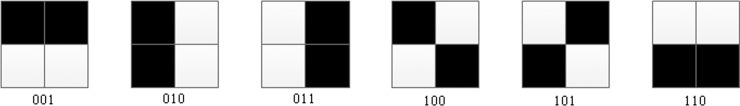
Image block classification and sub category encoding.

For the non-homogenous block, it is divided into four mutually non-overlapping sub-blocks *A*_*i*_ (*i* = 1, 2, 3, 4) of the same size, and each sub-block is 2×2. For each sub-block *A*_*i*_, the 11 bits feature information is generated, denoted as *F*_*i*_ = {*f*_*i* 1_, *f*_*i* 2_, …, *f*_*i* 11_}. where *f*_*i*1_ ~ *f*_*i*6_ are the binary encoding of the high six-bit average value of sub-block *A*_*i*_, namely

$$f_{i1}∼f_{i6}={\left(\left\lfloor \frac{1}{4}\sum_{j=1}^{4}\left\lfloor x_{i}{}_{j}/4\right\rfloor \right\rfloor \right)}_{B}$$ (14)

where *f*_*i*7_ ~ *f*_*i*9_ represent sub-category encoding. Fig 4 shows the six types and corresponding sub-category encoding of sub-block *A*_*i*_; the black represents the position of the maximum two pixels in the 2×2 image block.

*f*_*i*10_ ~ *f*_*i*11_ represent the binary value encoding of the difference between the sum of the maximum two pixels and the sum of another two pixels with uniform quantization, namely

$$f_{i10}∼f_{i11}={\left(\left\lfloor \frac{1}{32}((\left\lfloor x_{i1'}/4\right\rfloor +\left\lfloor x_{i2'}/4\right\rfloor )-(\left\lfloor x_{i3'}/4\right\rfloor +\left\lfloor x_{i4'}/4\right\rfloor ))\right\rfloor \right)}_{B}$$ (15)

In Formula (15), four pixels meet *x*_*i*1'_ ≥ *x*_*i*2'_ ≥ *x*_*i*3'_ ≥ *x*_*i*4'_

To enhance the security of the watermark, the recovery watermark must be encrypted. In this paper, the image sub-block size is used (if the block size is *N*×*N*, then *N* will be used as a random number) to generate two-value pseudo-random sequences *B*_*i*_ = {*b*_*ij*_|*j* = 1,2,⋯,*v*}. The recovery watermark *W*_*i*_ = {*w*_*i*1_,*w*_*i*2_,⋯,*w*_*iv*_} is encrypted.

$$w_{ij}=f_{ij}⊕b_{i}{}_{j},j=1,2,\cdots ,v$$ (16)

where, ⊕ is the XOR operation, *v* is the recovery watermark size of sub-block, and *v* = 11.

To increase the security of the authentication algorithm, each block is implemented by scrambling transform, that is, the corresponding recovery information of a block is mapped to other blocks according to the encryption key. The blocks around the tampered area are also easily tampered with, so a certain distance exists between the block and the mapping block.

### 3.2 Watermark embedding

**Fig 5:**
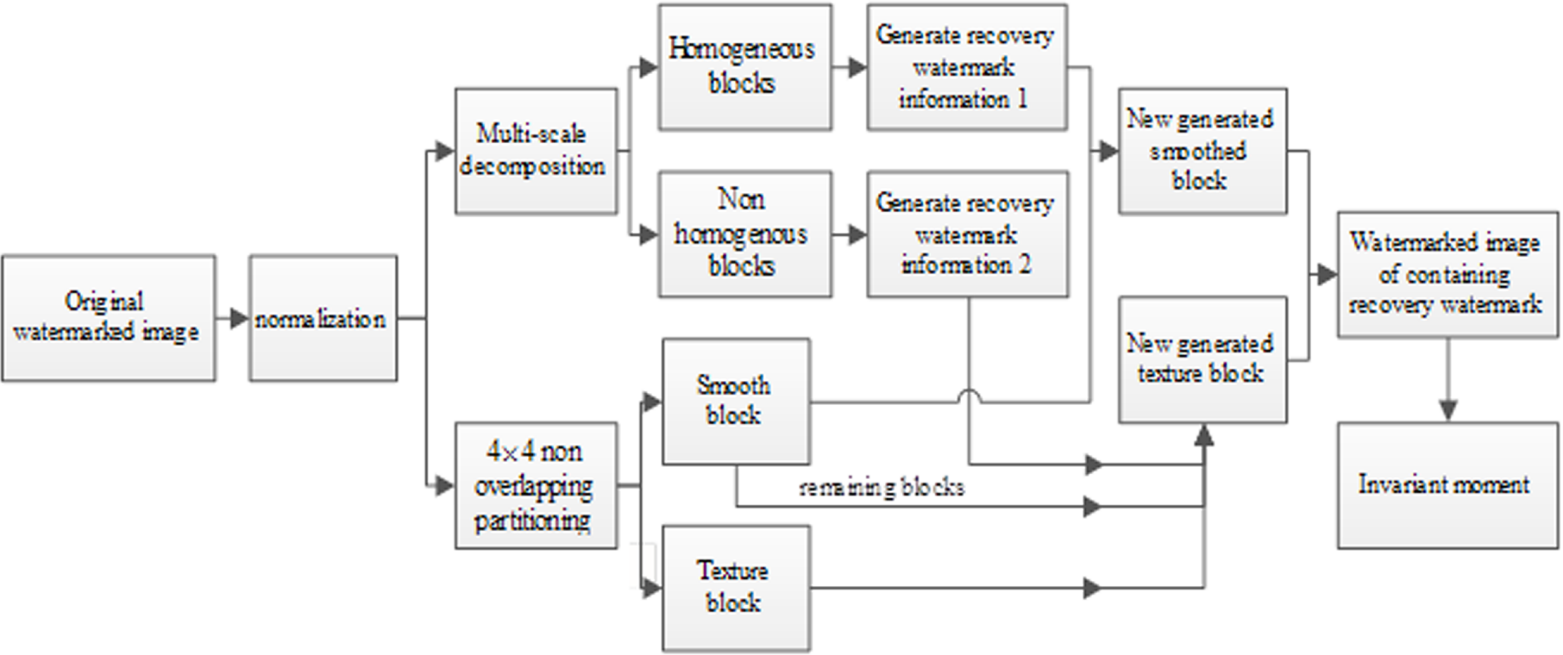
The flow chart of recovery watermark embedding.

The recovery watermark embedding process is shown in Fig 5, and the specific operation process is as follows:

(1) The original watermarked image is normalized (select circle)

The general image watermarking algorithm selects the whole image as the watermark embedding domain, but a rotation attack can lead to loss of image edge information in the realization process.

**Fig 6:**
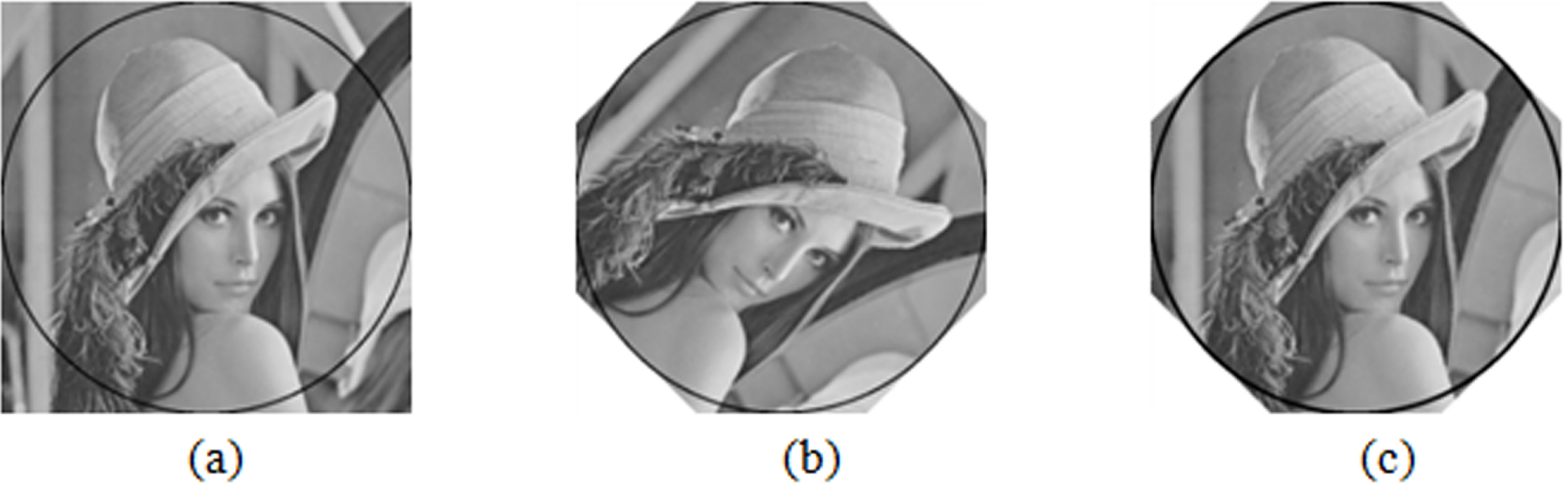
The four corner area information is lost after rotation. (a) represents original Lena image. (b) shows clockwise rotation of 45°C. (c) shows compensate for 45°C rotation.

As shown in Fig 6, information from the four corners of the obtained image is entirely lost after rotation and rotation correction. At this time, the corner information can be lost if the whole image is chosen as the watermark embedding domain when it is subjected to rotation attacks, resulting in reduced tamper localization precision. To solve this problem, we select the inscribed disc of the carrier image as the Zernike moments computational domain because the inscribed disc is the largest rotation invariant domain of the carrier image.

(2) After the normalization process, original image *I* is decomposed based on multiple scales, and the decomposed information (i.e., Huf(*q*) length and encoding table) is sent to the receiving party via the key method.

In this paper, image *I* is decomposed based on multiple scales, and the minimum block size is 4×4 after normalization processing. Therefore, the size of normalized image *I* should be extended to 4^*n*^×4^*n*^. The edge should add zero if the image is insufficient.

(3) Each sub-block *I*_*P*_ (0 ≤ *p* ≤ *N*, *N* is total number of sub-blocks obtained by decomposition of the original image) is sorted after decomposition. The original image *I* can be divided into homogeneous and non-homogenous image blocks, which can be further delineated into groups by multi-scale decomposition. From top to bottom and left to right, the sequences of homogeneous and non-homogenous image blocks are sorted, respectively, and the characteristic value of each ordered sub-block *I*_*P*_ is obtained as the recovery watermark. The recovery watermark will be encrypted after transformation into a two-value sequence. At the same time, sorting results are sent to the receiver by the key for use in image authentication or recovery.

**Fig 7:**
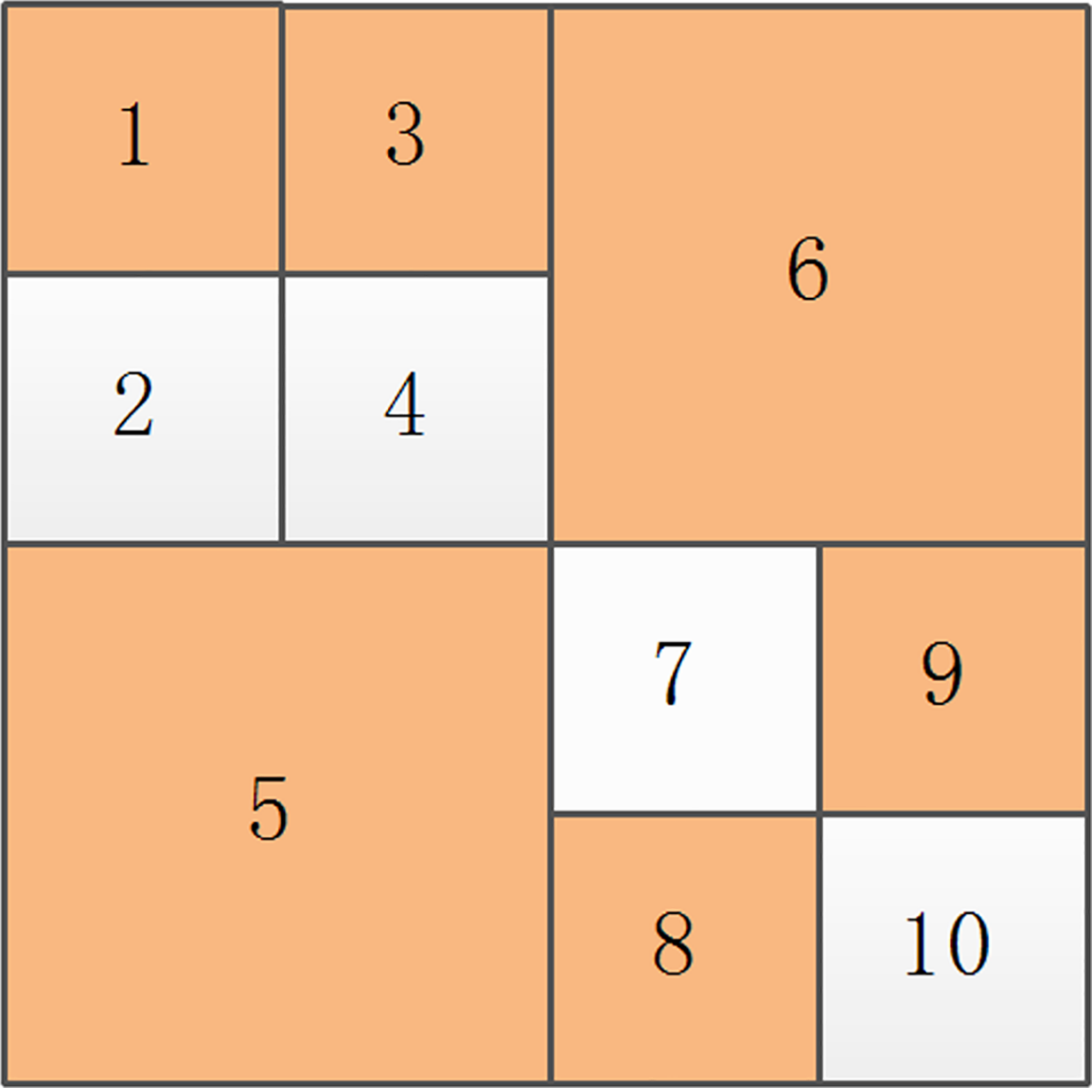
The multi-scale decomposition image.

Assuming the original image is decomposed by multi-scale decomposition, the resulting image is shown in Fig 7. For each sub-block number (Fig 7), the sorting result of the sub-blocks after decomposition is 1, 2, 3, 4, 5, 6, 7, 8, 9, and 10 from top to bottom and left to right. After the original image is decomposed, sub-blocks are divided into homogeneous and non-homogenous blocks. If the homogeneous image blocks are marked with orange (Fig 7), they will be 1, 3, 5, 6, 8, and 9; the sorting results of non-homogenous image blocks will be 2, 4, 7, and 10.

Based on the recovery watermark generating thought in Section 3.1, the average value of all pixels in a block is calculated as the recovery watermark of the homogeneous block, represented by an 8-bit binary number. Non-homogenous blocks are divided into four mutually non-overlapping sub-blocks *A*_*i*_ (*i* = 1, 2, 3, 4) of the same size, and each sub-block is 2×2. For each sub-block A_*i*_, the 11-bit feature information is generated as the recovery watermark of sub-block *A*_*i*_.

(4) After normalization, the watermarked image *I* is divided into non-overlapping sub-blocks *I*_*i*_ with a size of 4×4. The number of sub-blocks is *M*, *M* = $\frac{4^{n}\times 4^{n}}{4\times 4}$. The new recovery watermark information is generated by combining the generated recovery watermark according to Step 3 and the error-correcting code. The algorithm is also put into chaos to improve embedding security.

By calculating the entropy of each sub-block *I*_*i*_, all blocks are divided into the smooth area *I*_a_ and texture area *I*_b_ according to the set threshold entropy *T*_1_.

The entropy S of the gray-level co-occurrence matrix [19] is defined as:

$$S=-\overset{255}{\underset{i=0}{\sum }}\overset{255}{\underset{j=0}{\sum }}P(i,j)\mathrm{lg}P(i,j)$$ (17)

where *P*(*i*, *j*) is the value of the gray-level co-occurrence matrix *P* in (*i*, *j*).

**Fig 8:**
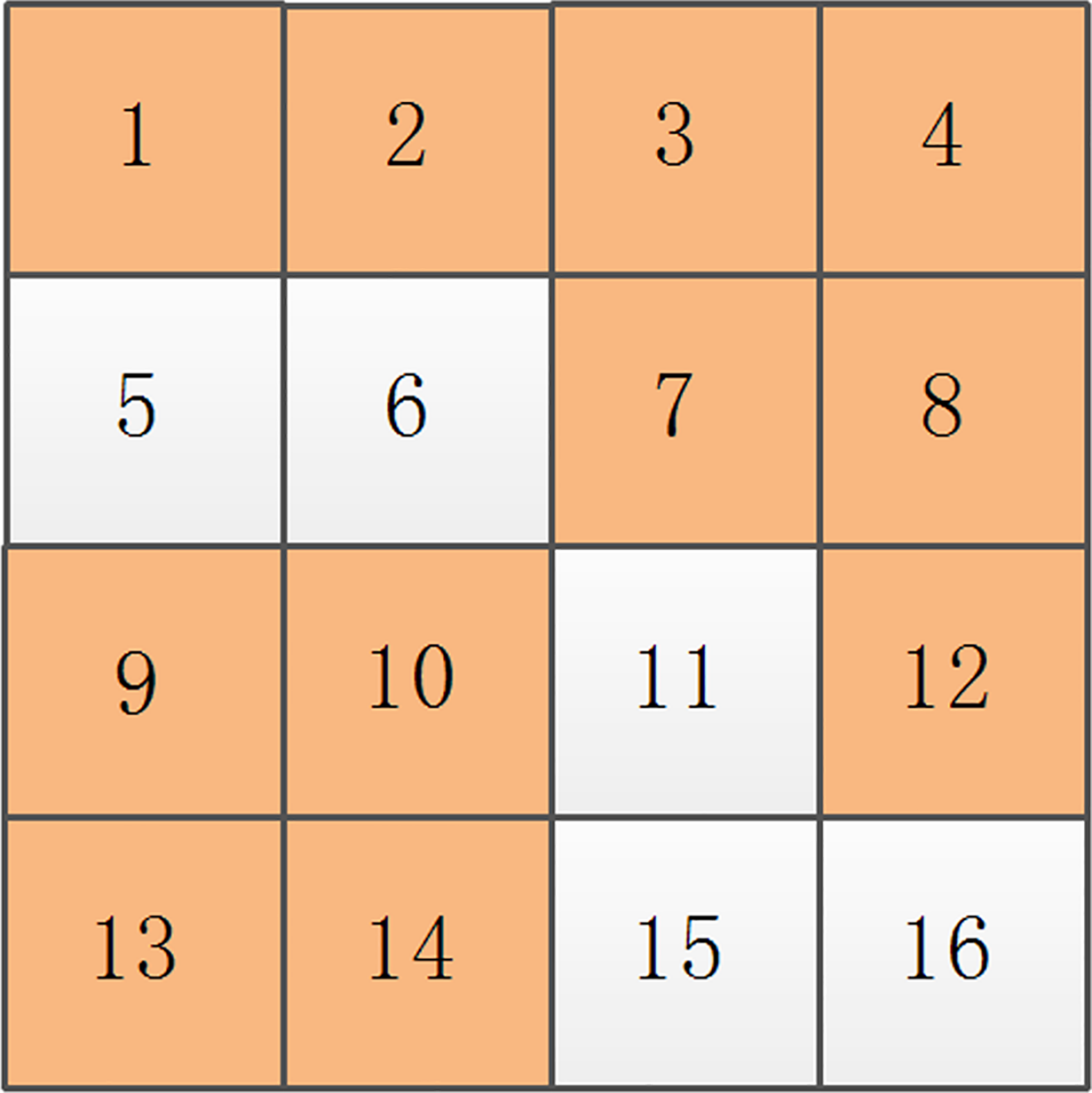
The 4×4 decomposition image of the original image.

Supposing that the original image is divided into 4×4 non-overlapping blocks, with block partitioning is shown in Fig 8. For each sub-block number (Fig 8), the sorting result of the sub-blocks after decomposition is 1, 2, 3, 4, 5, 6, 7, 8, 9, 10, 11, 12, 13, 14, 15, and 16 from top to bottom and left to right. By calculating the entropy of each sub-block, all sub-blocks are divided into smooth and texture areas. If the smooth blocks are marked with orange (Fig 8), the sorting result of the smooth blocks is 1, 2, 3, 4, 7, 8, 9, 10, 12, 13, and 14 and that of the texture blocks is 5, 6, 11, 15, and 16 from top to bottom and left to right.

Normally, homogeneous blocks corresponding to smooth blocks of 4×4 are obtained through multi-scale decomposition, equivalent to a homogeneous block being composed of one or more smooth blocks; the number of smooth blocks is far greater than that of homogeneous blocks. As a result, the recovered watermark information generated by homogeneous image blocks is embedded into corresponding smooth blocks, thus certainly producing redundant smooth blocks that have not been embedded with any watermark information. The remaining smooth blocks and texture blocks are combined to embed the recovered watermark information generated by the non-homogenous blocks.

(5) Using a logistic chaotic map to establish the mapping function between image sub-block *I*_*P*_(0 ≤ *p* ≤ *N*) and sub-block *I*_*i*_(0 ≤ *i* ≤ *M*), the watermark information generated by *I*_*P*_ is embedded into the corresponding mapping block *I*_*i*_. The key of the logistic chaotic map is saved to detect and extract the watermark information.

Based on key *K*_1_, the mapping function between the homogeneous image blocks and smooth blocks of the original image is established through the logistic chaotic map. Grounded on key *K*_2_, the mapping function among the non-homogenous image blocks, combined residual smooth block, and texture block is established through the logistic chaotic map.

(6) In this paper, the generalized difference expansion algorithm [20] and LSB algorithm [21] are applied to embed the recovery watermark produced by homogenous and non-homogenous image blocks into the corresponding*I*_*i*_, respectively.

In general, the smoother the blocks are, the smaller the difference between the internal pixels will be. After embedding the watermark information using the difference expansion method, the resultant objective distortion of the original carrier image is relatively small and can be a selection priority. Using the generalized difference expansion algorithm, the embedding watermark in smooth areas can effectively improve the embedding capacity and visual quality, but the pixel points beyond the range of the gray value are produced in the embedding process, so it is necessary to build an overflow map.

The overflow map is constructed after using the difference expansion algorithm to embed information; the pixel points beyond the scope of the gray value are marked in the map. Compressing the overflow graph, together with the recovery watermark generated by this homogeneous image block is embedded into the corresponding image smoothing block. The homogeneous image block generates 8 bits of watermark, and the algorithm divides smooth blocks into 4×4, so the generalized difference expansion can embed 15 bits binary number. In this paper, BCH (15, 11, 1) error-correcting code is used for encoding, which occupies 4 bits, hence the overflow information is embedded into the remaining 3 bits.

For the image block of size 4×4, the lowest two bits of pixels can be embedded with 32-bit information using the LSB algorithm. In this paper, the non-homogenous image block is divided into four equal-sized non-overlapping sub-blocks, each of which generates 11-bit recovery watermark and 4-bit error-correcting codes. Each corresponding sub-block must be embedded in 15-bit binary information. The recovery information generated by the two sub-blocks in the non-homogenous image block with the error-correcting code is embedded into the lowest two bits of the corresponding *I*_*i*_ mapping block.

The recovery watermark generated by a non-homogenous block needs to be embedded into two blocks *I*_*i*_, thus the non-homogenous blocks are divided into two same-sized non-overlapping blocks to be involved in the order of the original image by multi-scale decomposition when mapping between sub-blocks *I*_*P*_ and *I*_*i*_. Then the corresponding relationship between the two is established using a logistic chaotic map.

Assuming the sorting chart is obtained after multi-scale decomposition, and each block in the diagram corresponds to the order number.

**Fig 9:**
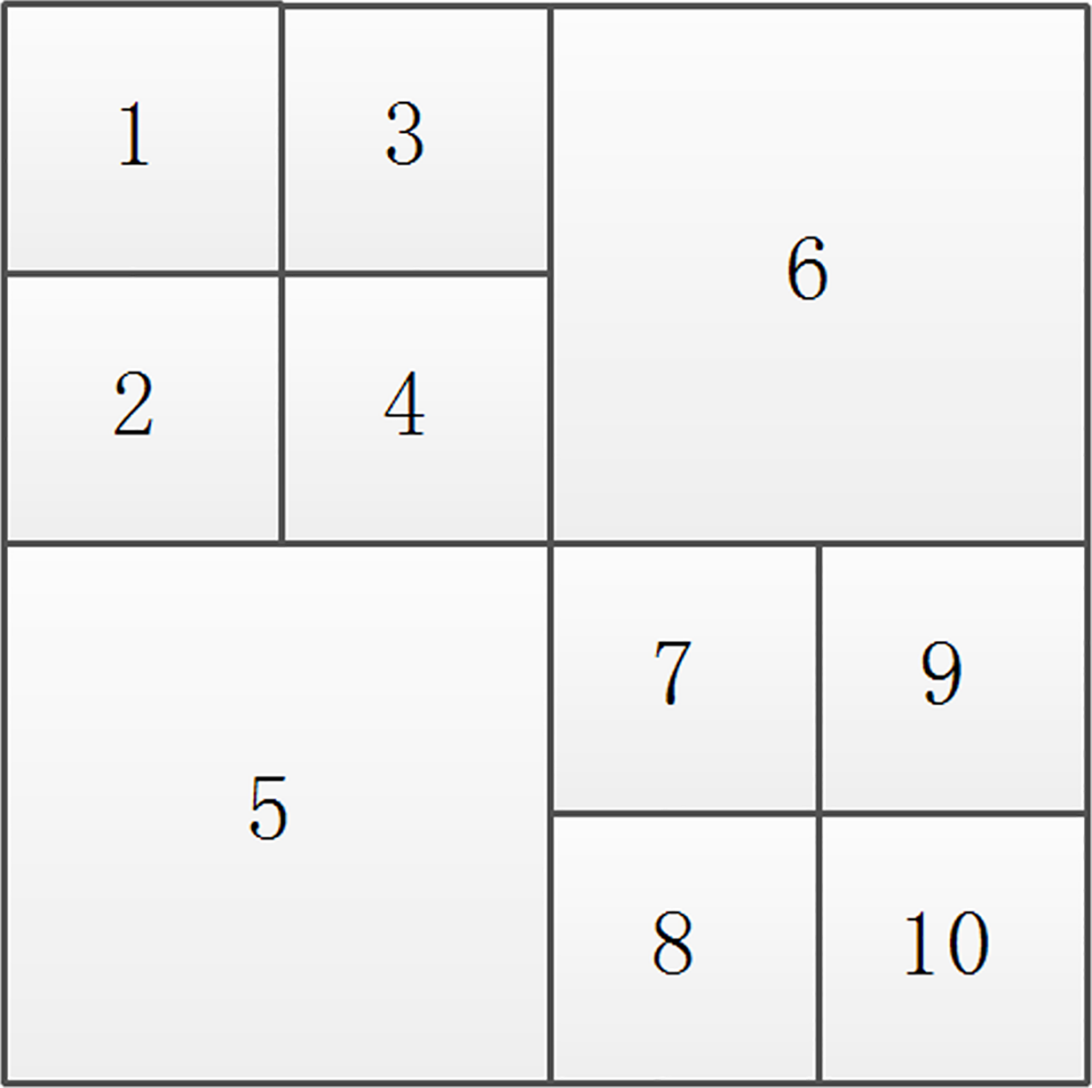
A sort graph obtained by multi-scale decomposition.

**Fig 10:**
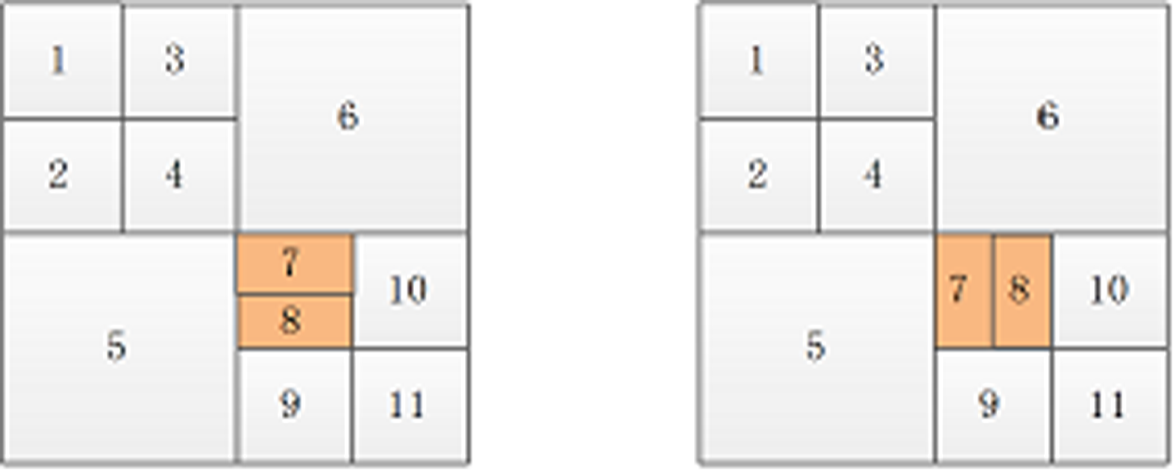
The new sort graph.

In Fig 9, if the smallest block after sorting as 7 is a non-homogenous block after multi-scale decomposition, then the new sorting chart (Fig 10) is as follows:

In this paper, the generalized difference expansion algorithm is used to embed the recovery watermark generated by the homogenous block into the smooth block area. The LSB algorithm is used to embed the recovery watermark information generated by the non-homogenous block into the redundant smooth blocks and texture blocks.

(7) The recovery watermark information generated by homogenous and non-homogenous blocks is embedded into the corresponding area, after which the image containing the recovery watermark can be obtained.

(8) The invariant distance of the embedded recovery watermark image is calculated.

### 3.3 Tamper localization and recovery

To calculate the invariant distance of the detection watermarked image, the geometric attack type is determined by comparing the geometric calibration parameter Para according to the image detection method introduced in the paper, which is then used to carry out geometric rectification of the detection image.

**Fig 11:**
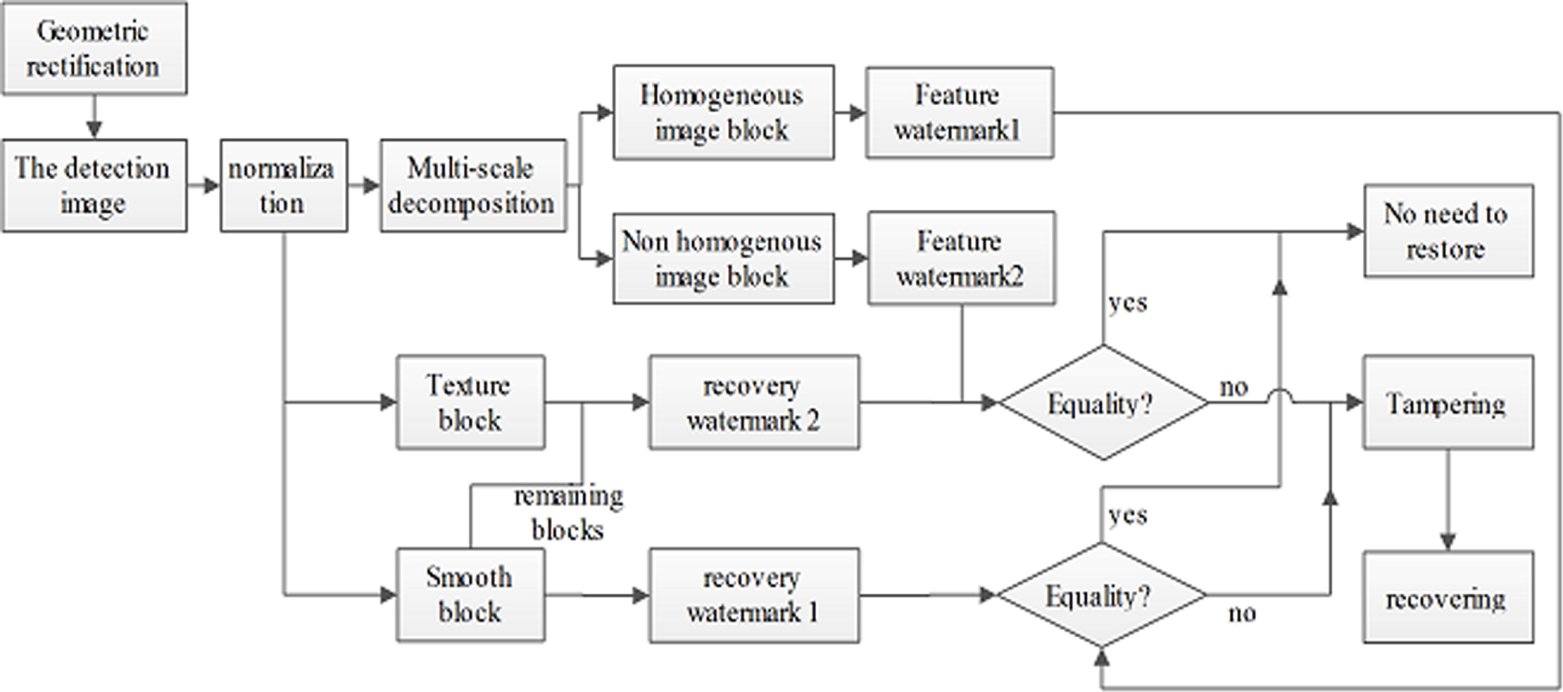
The flow chart of image tamper localization and recovery.

The process of tamper localization and recovery is shown in Fig 11, and the specific operation process is as follows:

1. The inscribed circle of the watermarked image after correction and normalization is selected as the extracting *w* domain;

2. The receiver receives the multi-scale decomposition information of the original image from the sender through a secret passage to carry out multi-scale decomposition of detection image *I*′.

3. After decomposition, every sub-block $I_{P}^{\prime }$ (0 ≤ *p* ≤ *n*, *n* is the total number of sub-blocks obtained after the detection image is decomposed) is sorted. The original image is decomposed by the multi-scale decomposition, and its sub-blocks can be divided into homogeneous and non-homogenous image blocks. To maintain consistency with the arrangement order of detection blocks and that of original image blocks and to prevent homogeneous blocks are tampered into non-homogenous blocks, and non-homogenous blocks are altered into homogeneous blocks when the detection image is attacked, the receiver receives the arrangement order of the original image block from the sender through the secret passage to classify and sort detection image block *I*′.

Based on the watermark generating thought in Section 3.1, the average value of all pixels in a homogeneous block is calculated as the authentication watermark of the homogeneous block, represented by an 8-bit binary number; the non-homogenous block is divided into four mutually non-overlapping sub-blocks *A*_*i*_ (*i* = 1, 2, 3, 4) with the same size (2×2). For each sub-block A_*i*_, the 11-bit feature information is generated as the authentication watermark of sub-block *A*_*i*_.

5. After normalization, the watermarked image *I*′ is divided into non-overlapping sub-blocks *I*_*i*_ with the size 4×4. The number of sub-blocks is *M*, *M* = $\frac{4^{n}\times 4^{n}}{4\times 4}$. By calculating the entropy of each sub-block *I*_*i*_ in the original image, all sub-blocks are divided into smooth area *I*_a_ and texture area *I*_b_. All sub-blocks in each class are sorted. To maintain consistency with the arrangement order of detection image blocks and that of original image blocks and to prevent smooth blocks are tampered into texture blocks, and the texture blocks are altered into smooth blocks, the receiver receives the arrangement order of the original image block from the sender through the secret passage to classify and sort detection image block *I*′.

6. Based on key *K*_1_, the mapping function between the homogeneous blocks and smooth blocks of the detection image is established using the logistic chaotic map. Based on key *K*_2_, the mapping function among the non-homogenous blocks and the combined residual smooth blocks and texture blocks is established using the logistic chaotic map.

7. The watermark information of smooth blocks corresponding to homogeneous blocks is extracted by the generalized difference extended inverse operation. The watermark information of the residual smooth blocks and texture blocks corresponding to the non-homogenous blocks is extracted using the LSB algorithm.

Image tampering may change the information embedded in sub-block *I*_*i*_. Hence, the information embedded in sub-block *I*_*i*_ must be corrected through BCH encoding to obtain more accurate recovery watermark information.

After deduction, a group of pixels is transformed by generalized difference expansion while the mean value of the group remains unchanged. The derivation process is not described in this paper due to length. Therefore, this paper tries to extract watermark information using the generalized difference expansion inverse operation. And 8-bit authentication information is obtained after decryption, error-correcting code error correction and overflow adjustment. The 8-bit authentication information and 8-bit authentication watermark generated in the corresponding homogeneous block are compared, and then the homogeneous blocks are judged whether they have been tampered with. The tampered homogenous block will be labeled.

Each non-homogenous block is divided into four sub-blocks, wherein each two sub-blocks are combined together to map to the residual smooth block and the texture block. The lowest two bits of each pixel in the block (residual smooth block and texture block) are extracted by the LSB algorithm to constitute authentication watermarks. The 22-bit feature information is obtained after the authentication watermark is decoded and corrected by the error-correcting code. The 22-bit feature information and corresponding 22-bit authentication watermark generated from two sub-blocks in non-homogeneous blocks are compared to determine whether each non-homogenous sub-block has been tampered with. The tampered non-homogenous blocks and its sub-blocks are labeled.

8. The marked tampered blocks are restored to ensure the integrity and practicality of the image. The recovery watermark is extracted in sub-block *I*_*i*_ corresponding to the tampered image block; the tampered area is recovered by the recovery watermark extracted from this block.

## 4 Experimental results and analysis

**Fig 12:**
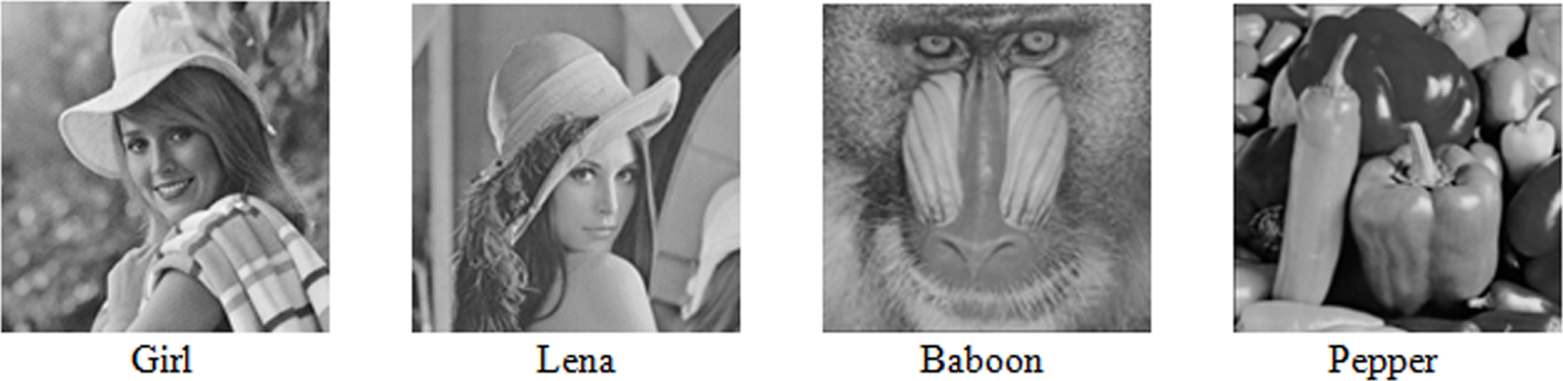
Original carrier images.

In this experiment, Girl, Lena, Baboon and Pepper images were selected as the original images, which are 512×512 standard images in 8-bit gray-scale (Fig 12). All images were obtained from http://sipi.suc.edu/database. The experimental environment was MATLAB 2012a in Windows XP. The Lena image serves as an example to verify the effectiveness of the algorithm. The processes and methods of other manipulated images are similar. The experiment emphasizes the reversibility of the algorithm, the visual quality of watermarked images, tamper detection precision and image restoration quality. The watermark capacity of the algorithm is variable. If the parameter *γ* value under multi-scale decomposition is different, then the partitioning of the original image is also different, meaning it has a different recovery watermark capacity. To analyze the algorithm performance more effectively, *γ* = 0.3 is taken. All the experimental results were achieved in Windows XP under the MatlabR2012a experimental platform. The experimental design is as follows:

1. Reversibility of the algorithm: the consistency of the restored image and original image is measured when the detection watermarked image has not been tampered with;
2. Visual quality of the watermarked image: the similarity between the generated recovery watermarked image and original image is measured after embedding the recovery watermark;
3. Tamper location accuracy: the location precision and detection accuracy of the tampered area are measured when the detection watermarked image is tampered with during tampering attacks;
4. Visual quality of the restored image: the similarity of the restored image and original image is measured to restore the located tampered area.

To ensure watermark embedding is consistent with the initial position, especially when the image is rotated, the edge angle information will be lost, decreasing the tamper localization precision. The inscribed disc of the carrier image is selected as the watermark embedding area to solve this problem. To calculate the invariant distance of the detection watermarked image, the geometric attack type is obtained through comparison with the geometric correction parameter Para. This geometric method of the detection image corrected by geometric correction can be used to ensure the robustness of watermarking.

### 4.1 Algorithm reversibility evaluation

Generally, the reversible watermarking algorithm requires the carrier image to recover completely after extracting watermark images. Therefore, it is measured by the Normalized Correlation (NC) of the original carrier image and the recovered carrier image after extracting watermark. The calculation formula is shown in Eq (18):

$$NC=\frac{\sum_{i=0}^{L-1}\sum_{j=0}^{K-1}I\left(i,j\right)I^{'}\left(i,j\right)}{\sum_{i=0}^{L-1}\sum_{j=0}^{K-1}{\left[I\left(i,j\right)\right]}^{2}}$$ (18)

where *I*(*i*,*j*), *I*′(*i*,*j*) respectively denote the pixel value at (*i*,*j*) of the original image and the recovered carrier image after extracting watermark. *L* and *K* respectively denote the rows and columns of the image. For the original carrier image and restored carrier image, the *NC* value must be 1, such that carrier images are generally required to recover completely.

In this paper, we use the generalized difference expansion algorithm and LSB algorithm to embed the recovery watermark into the corresponding area. When the containing recovery watermark image has been tampered with in the absence of tampering attacks, the recovery watermark is extracted and the original image is recovered. At this time, the correlation coefficient between the restored image and the original image is 1, which is *NC* = 1, showing that the algorithm can completely recover the original image and indicating the algorithm is reversible.

### 4.2 Watermarking image visual quality assessment

Currently, the peak signal to noise ratio (PSNR) is one of the main indicators for evaluating the visual quality of reversible watermarking. The greater the PSNR value is, the less the representative image distortion is, and the better the visual quality of watermarked images is. The smaller the PSNR value is, the more the representative image distortion is, and the more compromised the visual quality of watermarked images is. The calculation formula is shown in Eq (19):

$$PSNR=10\log\left(\frac{255^{2}}{\frac{1}{MN}\sum_{i=0}^{M-1}\sum_{j=0}^{N-1}{\left[I^{'}\left(i,j\right)-I\left(i,j\right)\right]}^{2}}\right)$$ (19)

*I*(*i*,*j*), *I*′(*i*,*j*) respectively denote the pixel value at (*i*,*j*) of the original image and the watermarked image. *M*, *N* respectively represent the rows and columns of the image. The visual quality is generally believed to be acceptable if the lost image quality is partial and the PSNR value of the visual quality of reconstruction is no lower than 30dB.

The realization of the whole algorithm should meet the basic requirements of the human visual system. In this paper, tamper detection and recovery algorithms are used to detect and recover the watermarked image when it has been tampered with by a small attack. If the watermarked image is damaged by a larger attack (the damage rate is 6%), the image is severely compromised; thus, its value is negligible and should be re-obtained. Hence, the algorithm determines whether an image suffers from a small or imperceptible attack by the human eye, which would suggest that fewer images have been tampered with.

According to Table 2, the PSNR value of the proposed algorithm is slightly higher than that of literature [22] and [3]. The 8-bit recovery watermark generated by homogeneous blocks (minimum 4×4 pixels) of the larger areas is embedded in the original smoothing blocks, and the visual quality is improved effectively. In literature [22], the PSNR of the generated watermarked image is slightly higher than that of literature [3] because of the "smoothing" technique used in the watermark embedded in literature [22].

**Table 2:** The visual quality evaluation of watermarked images under various attacks (dB).

attack modes	visual quality (PSNR)			
	this algorithm	Literature [22] algorithm		Literature [3] algorithm
no attack	41.34	40.43		36.67
Gaussian Filter (3×3 **σ** = 0.3)	37.47	37.27		32.91
Gaussian Filter (3×3 **σ** = 0.5)	36.03	35.53		31.64
median filtering [3,3]	37.17	35.66		32.77
adding white noise (0.01)	37.76	37.76		33.53
Salt and pepper noise (0.02)	37.04	36.43		32.78
shear (1/32)	30.17	29.11		24.92
JPEG Q = 20	35.65	35.31		31.92
JPEG Q = 50	38.21	36.72		33.57
shrink 30%	37.13	36.25		33.27
enlarge 30%	37.59	37.32		33.87
rotate 30°C	38.03	37.12		33.26
rotate 45°C	37.72	36.87		32.84

In this paper, the non-homogenous block of size 4×4 is divided into sub-blocks, and feature information is extracted from each 2×2 sub-block as the recovery watermark of each sub-block, which is able to increase the amount of recovery watermark generated by the non-homogenous block. The quality of the reconstructed image is thus improved.

In short, the watermark embedding capacity is fixed in literature [22] and [3], whereas it is variable in this paper. The digital image is smoother, the watermark embedding capacity is less, and the corresponding watermarked image quality is better. Thus, it provides a guarantee to improve the algorithm quality for improving the security and tamper recovery quality of the algorithm.

### 4.3 Tamper area detection and localization accuracy assessment

The watermarking embedding method is especially useful for determining the robustness and transparency of the entire digital watermarking system. A good algorithm can not only guarantee robustness, but also improve the accuracy of tamper detection.

The accuracy of tamper detection is calculated from two aspects as follows:

1. The positive detection rate (TPR), which is the ratio of the number of correctly detected tampering blocks to the total number of all tampering blocks;
2. The negative detection rate (TNR), which is the ratio of the number of inaccurately detected tampering blocks to the total number of all tampering blocks.

**Fig 13:**
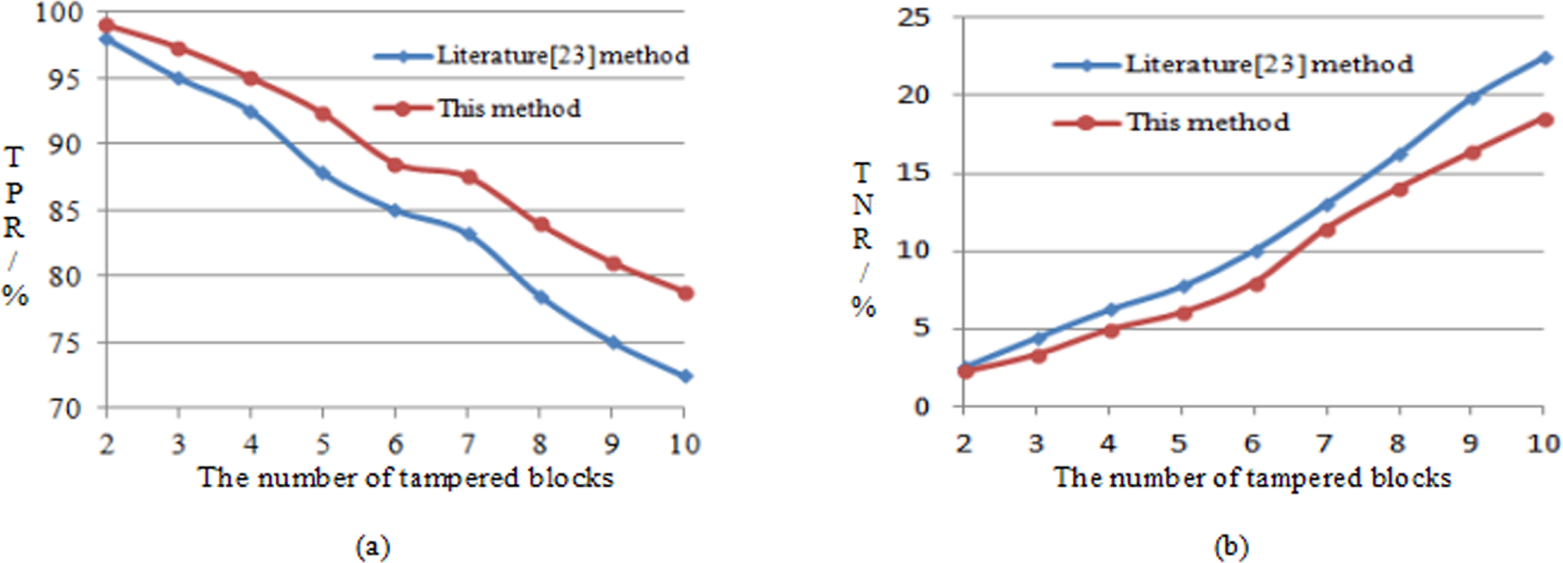
Comparison of the localization accuracy of the algorithm (40 experimental means). (a) represents comparison of positive detection rate. (b) shows comparison of negative detection rate.

Image tamper localization accuracy can be explained by a positive detection rate and negative detection rate. The pixel value of one or more pixel-blocks with a size of 32×32 of an image containing the embedded recovery watermark are randomly selected. When modifying, the average value of the block is unchanged, such as adding a gray level to half pixel, while the other half pixel minus a gray level. The detection results may differ from tampering at different locations of the same size in the same image. If two sub-blocks are tampered with and the location selection is different, the positive detection rate may change. Therefore, for the tampered area of two sub-blocks, the average positive detection rate is obtained by 40 experiments for different locations. Fig 13(A) shows the mean of TPR after 40 experiments; Fig 13(B) shows the mean of TNR after 40 experiments.

Fig 13(A) shows that the positive detection rate of the two methods exhibits a downward trend as the tampered blocks increase, but this method is clearly better than literature [23]; Fig 13(B) shows that an increase in tampered blocks leads to a negative detection rate, which demonstrates an upward trend. This method rises slowly relative to that in literature [23]. If tampering leads to information errors in the decomposition blocks, TNR will rise sharply. According to the comprehensive analysis of the curve data in Fig 13, the tamper localization accuracy of this method can be increased by nearly 4% compared with that in literature [23].

### 4.4 Restoration of image visual quality

The greater information capacity of original image contains in a recovery watermark (referred to as "watermark information capacity"), the greater the tamper detection probability of the algorithm is and the higher the tamper recovery quality is. An ideal condition is to use the smallest bits possible to preserve as much image information as possible.

**Fig 14:**
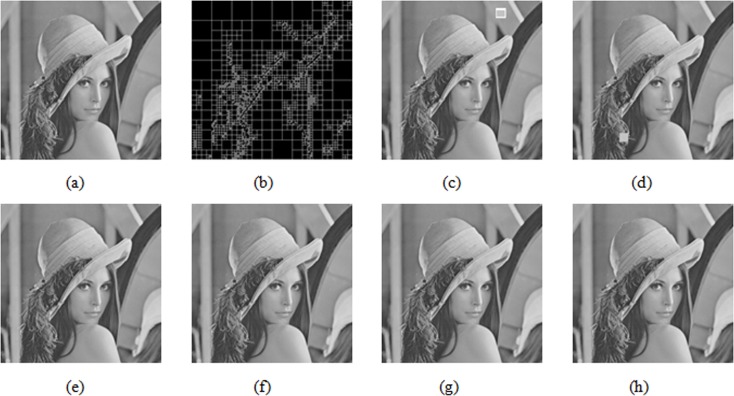
The tamper location and recovery results of the Lena images.

**Fig 15:**
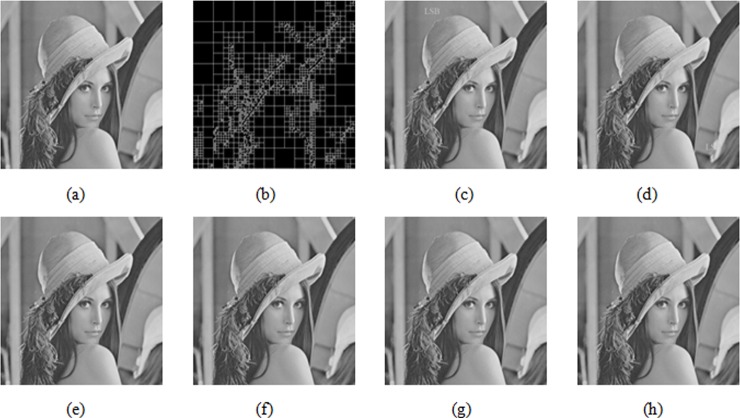
The tamper location and recovery results of the Lena images.

To prove the superiority of the proposed algorithm in feature value selection, the quality of the reconstructed image in the tampered area is compared with the Kim method [22]. Experimental results appear in Fig 14 and Fig 15. Fig 14(A) represents the original Lena image. Fig 14(B) represents the multi-scale decomposed image. Fig 14(C) represents the watermarked image of tampering in the area (362, 24, 32, 32), *PSNR* = 36.07dB. Fig 14(D) represents the watermarked image of tampering in the area (146, 427, 24, 24), *PSNR* = 38.10dB. Fig 14(E) represents the image after using Kim’s method to restore the tampered area of the image in Fig 14(C), *PSNR* = 58.72dB. Fig 14(F) represents the image after using the proposed algorithm to restore the tampered area in Fig 14(C), *PSNR* = 58.76dB. Fig 14(G) represents the image after using Kim’s method to restore the tampered area of the image in Fig 14(D), *PSNR* = 60.13dB, Fig 14(H) represents the image after using this algorithm to restore the tampered area of the image in Fig 14(D), *PSNR* = 75.66dB.

Fig 15(A) represents the original Lena image, Fig 15(B) represents the multi-scale decomposed image, Fig 15(C) represents the watermarked image of tampering in the area (288, 112, 32, 68), *PSNR* = 34.15dB, Fig 15(D) represents the watermarked image of tampering in the area (192,352,32,68), *PSNR* = 35.81dB, Fig 15(E) represents the image after using Kim’s method to restore the tampered area of the image in Fig 15(C), *PSNR* = 56.73dB, Fig 15(F) represents the image after using this algorithm to restore the tampered area in Fig 15(C), *PSNR* = 56.84dB, Fig 15(G) represents the image after using Kim’s method to restore the tampered area of the image in Fig 15(D), *PSNR* = 59.54dB, Fig 15(H) represents the image after using this algorithm to restore the tampered area of the image in Fig 15(D), *PSNR* = 74.33dB.

In Fig 14(C), the tampered area in the image is relatively smooth. (362, 24, 32, 32) represents a 32×32 image block starting from the coordinate point (362, 24). Since using the Kim method and this algorithm, eigenvalues obtained in the smooth area are nearly identical, and image quality after restoration with two methods is basically the same as shown in Fig 14(E) and 14(F). In Fig 14(D), however, the tampered area occurs in a more complex area (146, 427, 24, 24), and the restored image quality is greatly different. From the PSNR value in Fig 14(G) and 14(H), the proposed algorithm extraction is obviously superior to the average algorithm in the extraction of the recovery feature watermark in non-homogenous blocks, because the extracted recovery watermark considers the texture characteristics of the image block.

Similarly, in Fig 15, when the watermarked image is tampered with via another attack, the visual quality of the image after restoring the tampered image through this algorithm is better than the Kim algorithm.

The traditional image authentication watermarking algorithm can generate an authentication watermark based on block mean, whose position accuracy is high; however, it can’t resist a mean attack proposed in literature [24]. In this paper, the variable capacity authentication watermarking not only expands the amount of watermark information, but also improves the ability of the algorithm to resist the mean attack.

**Fig 16:**
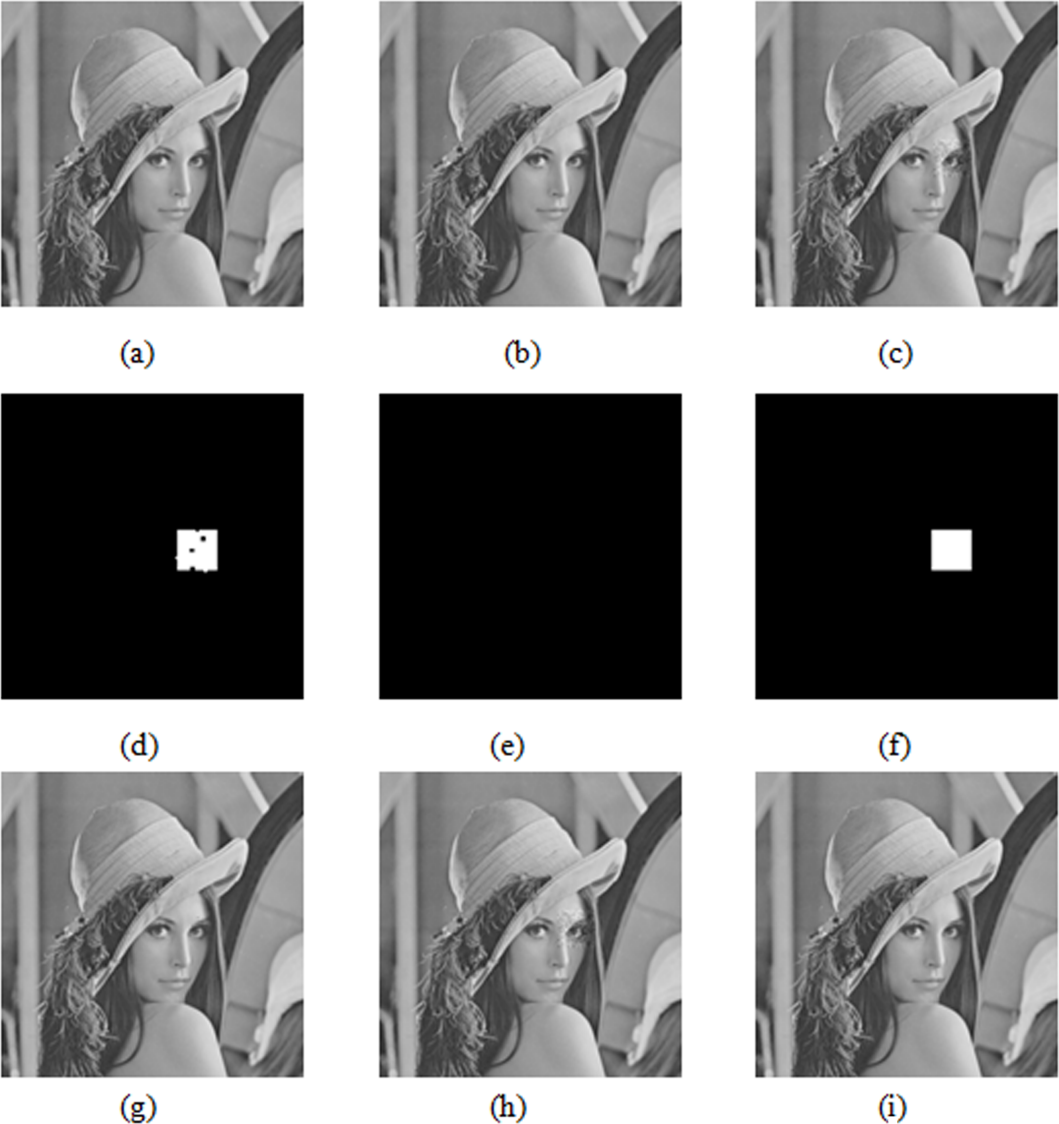
Comparison of tampering detection results and tampering recovery results under mean attack.

Fig 16 shows the tamper detection results of this paper and literature [3] and [25] under the mean attack. Fig 16(A) displays the Lena original gray image of size 512×512; Fig 16(B) shows the generated watermarked image using this algorithm; and Fig 16(C) is the tampering image, in which the square area of size 64×64 suffers a mean attack at a tampering ratio of approximately 1.56%. Fig 16(D), 16(E) and 16(F) display the results of the tamper detection in this paper and literature [3] and [25], respectively. Fig 16(G), 16(H) and 16(I) show the results of tamper recovery in this paper and literature [3] and [25], respectively.

As shown in Fig 16, literature [3] does not detect the tampered blocks under a mean attack. Hence, literature [3] does not perform tamper the recovery operation, implying that literature [3] can’t resist a mean attack. All tampered blocks are detected by literature [25], which can therefore effectively resist a mean attack with high positioning accuracy. In literature [25], however, tampering causes some encoding bits in the third layer to fail to be restored correctly, leading to somewhat poor restored image quality. The PSNR of the restored image is 54.46dB. In this paper, the tampered areas can be located mostly accurately using the variable capacity authentication watermark and the dual authentication algorithm, suggesting that the algorithm can effectively resist mean attacks. The PSNR of the restored image is 69.81dB using this algorithm.

**Fig 17:**
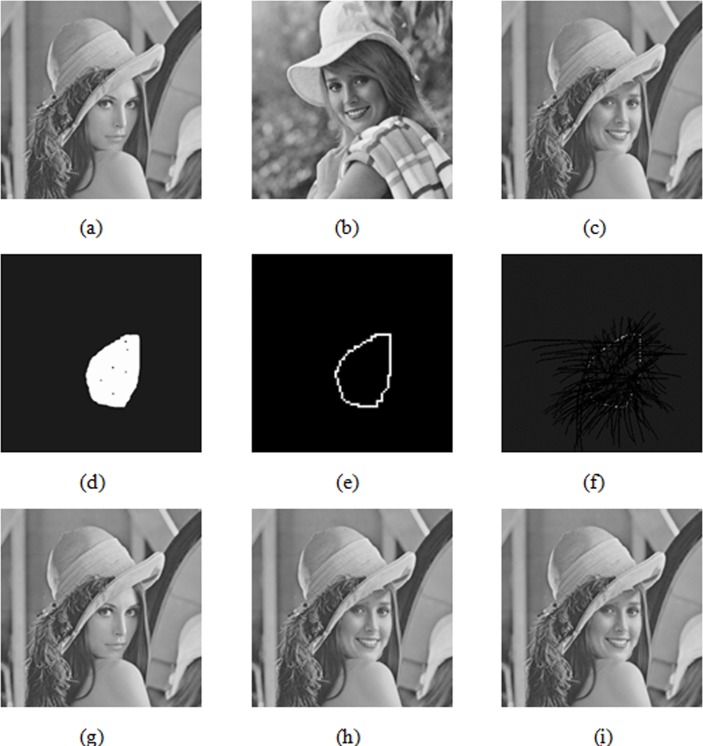
Comparisons of tampering detection results and tampering recovery results under collaging attack.

A collage attack is one of the greatest threats to the fragile watermarking algorithm [26], and many existing algorithms can't resist attacks effectively. Fig 17(A) shows a generated watermarked image of size 512 × 512 based on this algorithm. Fig 17(B) shows a generated watermarked Girl image of size 512 × 512 based on this algorithm. After replacing the head area of the watermarked Girl image to the same area (collage attack) with the watermarked Lena image, the tampered image is shown in Fig 17(C) at the ratio of approximately 13%. Fig 17(D), 17(E) and 17(F) illustrate the tamper detection results in this paper and literature [3] and [25], respectively. Fig 17(G), 17(H) and 17(I) present the tamper recovery results in this paper and literature [3] and [25], respectively.

As displayed in Fig 17, literature [3] and [25] only detect the boundaries of the collage area in the collage attack condition, leaving the interior of the collage area authenticated. Literature [3] and [25] can detect the collage area border of the image blocks, mainly because the collage area is not tampered with the image block as a unit. Because the missing tampered block does not perform tamper recovery operations, literature [3] and [25] have low tamper recovery quality. Their PSNR of restored images are 25.16dB and 25.72dB, respectively, indicating that these methods are not effective against collage attacks. In this paper, the tampered collage areas can be located accurately and the PSNR of the restored image is 63.82dB, suggesting that the algorithm can effectively resist collage attack.

Compared with literature [27] and [28], this algorithm also has certain performance advantages. Literature [27] proposed a self-recovery scheme for tampered images using VQ indexing and image inpainting. The recovery-bits of each cover block are generated by its VQ index and recovery-bits are embedded into the LSB planes of the cover image to produce the watermarked image. The algorithm has high recovery quality, but it cannot effectively resist geometric attacks such as mean attack, collage attack and so on. Literature [28] proposed an effective tamper detection and self-recovery algorithm based on singular value decomposition. A random block-mapping sequence and three unique optimizations are employed to improve the efficiency of the proposed tamper detection and robustness against various security attacks, such as collage attack and constant-average attack. The algorithm can effectively resist geometric attacks such as mean attack and collage attack, but the restoration quality necessitates further improvement. In addition, the algorithms in literature [27] and [28] do not belong to the reversible image recovery watermarking technologies, so they can not completely restore the original image without any attacks. Compared with the algorithm in this paper, the recovery effect is sub-optimal.

In this paper, the authentication recovery watermark capacity is variable. In other words, the smoother the relative digital image is, the less the amount of generated authentication watermark is. Under the premise of providing the appropriate amount of block information, the capacity of the authentication recovery watermark is reduced while the quality of the watermarked image is improved.

At the same time, the algorithm with high security and good tamper recovery quality can accurately locate the tampered location, detect tampered blocks, and obtain high-quality restored images under general tampering, mean attack and other attacks.

## 5 Conclusion

The integrity authentication of images is of great significance in the network environment. The image tampering detection method based on self-recovery has obvious advantages. The main contributions of this paper are as follows:

1. Since the fixed block divided method is not suitable for image tamper detection and high-quality recovery, this paper presents a method of multi-scale decomposition of the original image based on image texture characteristics to effectively overcome the defects of the fixed block division method. This method not only takes into account the characteristics of the image block area, but also has a great advantage in the embedding capacity and tamper detection precision.
2. Considering watermark embedding capacity and security, this paper proposes a new image watermarking method with variable recovery watermark capacities. Based on the multi-scale decomposition of the original image, the image block content feature is extracted to generate a variable capacity recovery watermark—homogeneous block 8 bits, non-homogenous block 44 bits. Variable capacity recovery watermark can preserve sufficient image block information with a smaller number of bits. It only involves one-time embedding for tamper detection and recovery, effectively reducing the watermark embedding capacity, while improving the visual quality of the watermarked image and the ability of the algorithm to resist mean attacks.
3. Using the generalized difference expansion algorithm, the recovery watermark generated by homogeneous blocks is embedded into the corresponding smooth blocks using the logistic chaotic map. With the LSB algorithm, recovery watermark information generated by non-homogenous blocks is embedded into the corresponding redundant smooth non-embedded blocks and texture blocks by logistic chaotic mapping. The watermarking embedding method effectively improves the embedding capacity and visual quality and resists the impact of collage attacks.
4. The corresponding watermark information embedded in the image can also be changed when the watermarked image is attacked. The main information of the image block can also change as the recovery watermark information changes, further influencing the original image content. In this paper, the error-correcting code is used to ensure the correctness of the recovery watermark data at the cost of sacrificing effective embedding capacity.
5. To offset watermark synchronization errors caused by geometric attacks, a geometric transformation of the watermarked image is estimated using the invariant moments of the image. Image normalization is also performed before recovery watermark embedding and in the image tamper location and recovery to avoid the influence of geometric attacks.

According to the experimental simulation results, the proposed algorithm with high tamper localization precision and good qualities of the obtained watermarked image and the restored image can effectively resist known forgery attacks such as collage attack, mean attack and so on. Subsequent research will explore how to improve tamper detection precision and recovery quality and reduce the embedding rate.
